# Impact of Chorionicity in Neurodevelopmental Outcomes in Preterm Twins

**DOI:** 10.7759/cureus.75029

**Published:** 2024-12-03

**Authors:** Catarina Leuzinger Dias, Catarina Cordeiro, Margarida Camacho-Sampaio, Andreia Lomba, Adelaide Taborda

**Affiliations:** 1 Neonatology Department, Maternidade Bissaya Barreto, Unidade Local de Saúde de Coimbra, Coimbra, PRT

**Keywords:** chorionicity, neonatology, neurodevelopment, prematurity, twins

## Abstract

Introduction

Multifetal pregnancies, which account for 2-4% of births worldwide, have increased in recent years. Twin pregnancies carry a higher risk of preterm birth and associated neonatal morbimortality, with monochorionic twins considered at greater risk. This study investigates the influence of chorionicity on neurodevelopmental outcomes in preterm twins.

Methods

A retrospective cohort study was conducted, including preterm twins born before 32 weeks of gestational age and/or with a birth weight of less than 1500 grams, admitted to a tertiary-hospital neonatal intensive care unit from 2013 to 2021. Neurodevelopmental outcomes were evaluated at 24 months of corrected age using the Griffiths II Mental Development Scales. Moderate to severe neurodevelopmental impairment was determined by the occurrence of one or more of the listed criteria: global development quotient <70, severe visual impairment, cerebral palsy, or profound sensorineural deafness.

Results

A total of 125 preterm twins were evaluated, of which 45% (n=56) were monochorionic. Overall, 5.6% (n=7) of the infants had moderate to severe neurodevelopmental impairment (NDI), with higher comorbidity rates in this group. No significant differences were found in NDI or other prematurity-related comorbidities between monochorionic and dichorionic twins. Gestational age over 27 weeks and birth weight over 1010 grams were identified as accurate predictors for an absence of moderate to severe NDI in these infants.

Conclusion

Chorionicity alone does not appear to independently affect neurodevelopmental outcomes in preterm twins when complications are effectively managed. Improved prenatal monitoring and appropriate treatment of twin pregnancies, especially monochorionic, are crucial to mitigate risks associated with moderate to severe neurodevelopmental impairment.

## Introduction

Multifetal pregnancies, accounting for approximately 2-4% of all births worldwide, have seen an increased incidence in recent decades. This has been attributed to wider access to assisted reproductive technology and a delay in maternal childbearing age [1-3].

Compared with singleton pregnancy, multifetal gestations carry significantly higher risks of maternal and fetal morbidity and mortality [1,4]. Twin pregnancies have a higher rate of preterm birth, which is widely recognized as a factor in perinatal mortality and neurodevelopmental complications [5,6]. The question remains whether the increased risks and poorer outcomes observed in preterm twins are primarily due to chorionicity or the effects of prematurity itself [7].

Chorionicity’s impact on infants' outcomes remains debatable, lacking consensus in current literature. Monochorionicity is associated with approximately double the risks of preterm birth, stillbirth, and neonatal complications [4]. Monochorionic twins (MC-T) are thought to be at the greatest risk for a suboptimal outcome [8,9] with a bigger likelihood of perinatal mortality and neonatal intensive care unit admission compared with bichorionic twins (BC-T) [10,11].

While survival rates in multifetal gestations are relatively well-documented, reports on factors contributing to neurodevelopmental impairment (NDI) remain scarce and inconclusive. Some authors report cerebral palsy (CP) rates 5 to 10 times higher in twins [12,13] but others showed that there was no significant difference in neurodevelopmental outcomes between twins and singletons [3]. Developmental delays, rather than survival rates themselves, emerge as a paramount concern, making the neurodevelopment assessment of these infants essential [14]. Identifying characteristics of multifetal pregnancies associated with heightened risk of NDI could be a first step to initiating targeted intervention and surveillance programs [15].

We hypothesized that chorionicity does not influence NDI in preterm twins. The main aim of this study was to assess NDI in premature MC-T and compare them to BC-T. Our secondary aim was to identify the risk factors for such outcomes.

## Materials and methods

Study design, patient selection, and data collection

We carried out a retrospective cohort study that included all twin births occurring <32 weeks of gestational age (GA) and/or with a birth weight (BW) of less than 1500 grams admitted at a tertiary-level maternity hospital neonatal intensive care unit (NICU) between January 2013 and December 2021. Neonates born with significant congenital malformations or genetic disorders were excluded from the study. Patients’ clinical data and follow-up information were obtained by revising medical records using the NICU’s database.

Prenatal and postnatal data

Data regarding maternal health and demographic factors were collected, including age, number of previous pregnancies, and delivery history. 

The perinatal factors evaluated included type of birth (cesarean or vaginal) and the administration of antenatal corticosteroids (ACT) to induce fetal pulmonary maturation (considered if the neonate had received four doses of dexamethasone or two doses of betamethasone). 

Neonatal characteristics and morbidity were also assessed: the need for advanced resuscitation at birth; newborn sex; gestational age (GA) at birth; and birth weight (BW) - a neonate was considered small for gestational age (SGA) when BW<10th percentile, based on the Fenton growth charts [16]. Neonatal sepsis was contemplated as clinical sepsis accompanied by abnormal laboratory results (leukocytosis or leukopenia, elevated C-reactive protein, or thrombocytopenia), regardless of blood cultures [17]. Necrotizing enterocolitis (NEC) was considered when ≥ stage 2 based on the Modified Bell’s staging system [18]. Bronchopulmonary dysplasia (BPD) was considered when oxygenotherapy was required at 36 weeks postmenstrual age [19]. Retinopathy of prematurity (ROP) was categorized according to the International Classification of ROP, and considered ≥ stage 2 [20]. Cystic periventricular leukomalacia (PVL) was defined as≥ grade 2 as per the De Vries et al. grading system [21]. Peri-intraventricular hemorrhage (PIVH) was graded as severe when categorized as ≥ grade III or in the presence of hemorrhagic infarction, following Volpe’s classification [22]. The need for invasive mechanical ventilation (IMV) and postnatal surfactant administration was also assessed.

Follow-up data

The institution’s protocol defined that all very preterm infants should be assessed in a follow-up program up to 36 months of corrected age, performed by a dedicated team, comprising pediatricians, nurses, and trained educators, with frequent reevaluations after discharge, especially at developmental milestones. 

A formal neurodevelopment evaluation was performed at 24 months of corrected age using the validated Griffiths Mental Developmental Scales II (GMDS-II) to calculate patients’ global development quotient (GDQ) and development quotients for specific areas, including locomotor skills, personal-social behavior, hearing and language, hand-eye coordination, and performance [23].

Cerebral palsy was diagnosed based on the definition and guidelines of the Global Motor Function Classification System (GMFCS) and the European Cerebral Palsy Network [24]. Hearing and vision deficits were consistently and thoroughly assessed by otolaryngologists and ophthalmologists. 

A child was considered to have moderate to severe NDI when at least one of the following criteria was met: GDQ<70 for GMDS-II; severe visual impairment (complete vision loss or no useful vision); CP; sensorineural deafness necessitating hearing device.

Statistical analysis

Version 29 of IBM SPSS Statistics® (IBM Corp., Armonk, NY, USA) was used to statistically analyze the collected data. Continuous variables are presented as means and standard deviations (SD) or median and interquartile range (IQR) according to their distribution. Categorical variables are presented as frequencies and percentages. Normal distribution was verified through the Kolmogorov-Smirnov test.

The comparative analysis of categorical variables was performed using either the χ2 test or Fisher’s exact test, depending on the suitability of the data. For non-parametric variables, the Mann-Whitney U test was employed to assess differences. An odds ratio (OR) with a 95% confidence interval (95% CI) was determined.

Logistic regression adjustment was executed to determine the predictors of moderate to severe (NDI). The quality of fit was assessed using the Hosmer and Lemeshow test while model significance was evaluated with the Omnibus test. A model was developed by adjusting for statistically significant and pertinent perinatal factors.

In addition, receiver operating characteristic (ROC) curves were performed to evaluate the accuracy of the GA and BW in predicting outcomes. The power of predictors to discriminate between outcomes was quantified by the area under the curve (AUC) of the ROC curve.

A two-tailed p-value of <0.05 was considered statistically significant for all performed analyses. The study was approved by the institution’s Ethics Committee (protocol number PI OBS.SF.207-2023).

## Results

**Figure 1:**
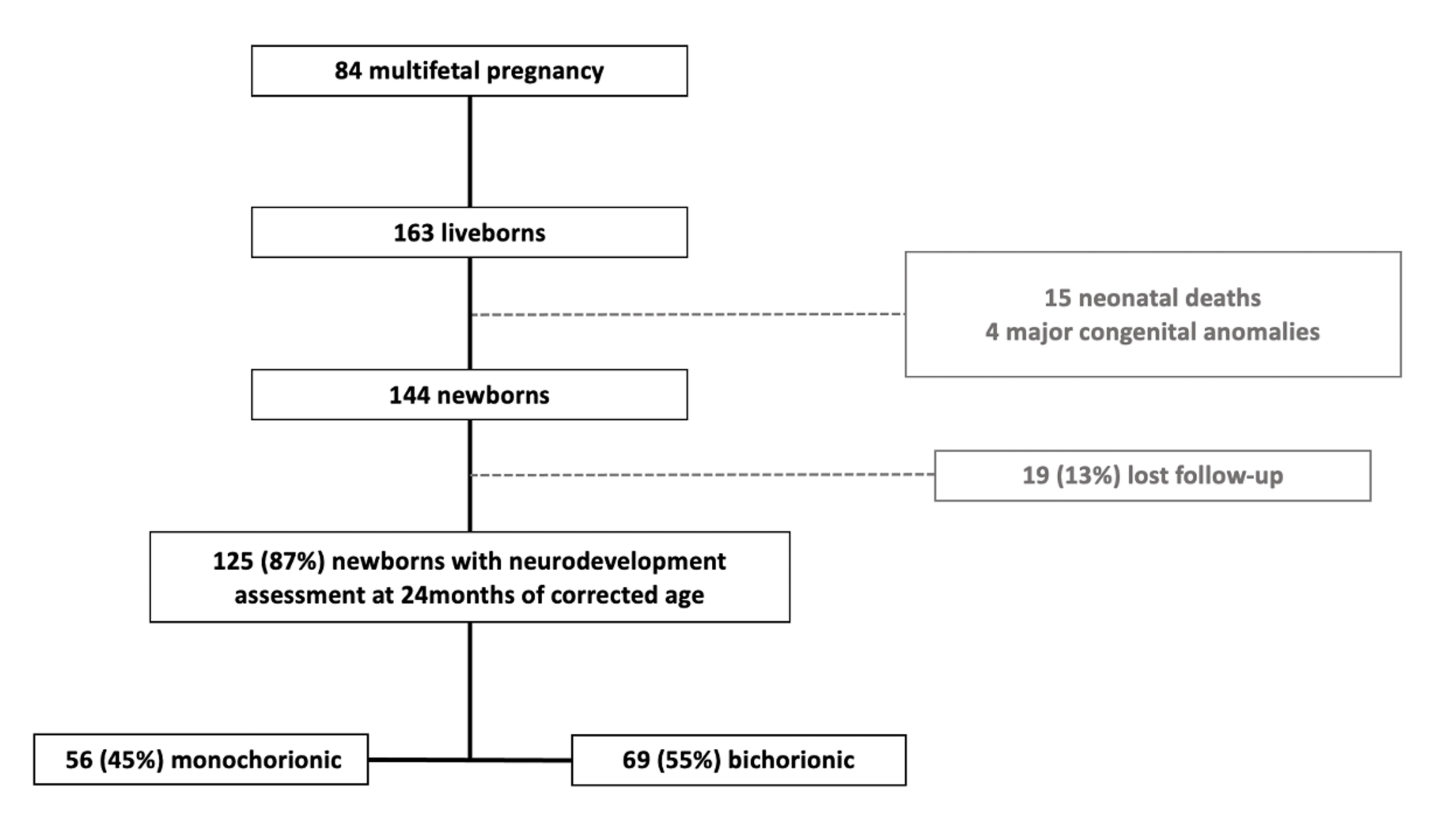
Flowchart of patient selection

Throughout the period of nine years, 84 multifetal pregnancies were determined as eligible, with 163 live births. Of these, four were excluded due to major congenital malformations and 19 were lost to follow-up. There were 15 (9%) neonatal deaths and a follow-up rate of 87% (n=125). The total study cohort consisted of 125 infants with neurodevelopment assessment at 24 months of corrected age. Regarding chorionicity, BC-T represented 55% (n=69) of our cohort. The flowchart of patient selection is shown in Figure 1.

Both MC-T and BC-T have a median GA of 31 weeks. There was a higher proportion of extreme prematurity among BC-T (p=0.034). This group also presented a lower rate of cesarean deliveries (p=0.010). No differences were found in BW between both groups or in other evaluated perinatal factors (Table 1). Among MC-T, 27% (n=15) had twin-twin transfusion syndrome (TTTS).

**Table 1:** Characteristics and comparison of infants according to chorionicity ACT – antenatal corticosteroid therapy; BPD – bronchopulmonary dysplasia; BW – birth weight; CPL – cystic periventricular leukomalacia; GA – gestational age; IMV – invasive mechanical ventilation; IQR – interquartile range; NDI – neurodevelopment impairment; NEC – necrotizing enterocolitis; PIVH – periventricular-intraventricular hemorrhage; ROP – retinopathy of prematurity; SGA – small for gestational age Categorical variable data are expressed in frequencies (n) and percentages (%) and analyzed using *χ² test or **Fisher’s exact test, as appropriate, with respective odds ratio. The test value is Fisher’s exact test, which is not displayed, as it is not applicable. Continuous variable data are expressed in median and interquartile ranges and analyzed using the #Mann-Whitney U test. Statistical significance was considered when p-value <0.05.

	MC-T (n=56)	BC-T (n=69)	Test statistic	p-value*	OR (CI 95%)
GA (weeks), (median | IQR)	31 | 2	31 | 4	2195.5	0.184^#^	
GA <28weeks, n (%)	5 (9)	16 (23)	4.497	0.034^*^	0.33 (0.11-0.95)
BW (gram), (median | IQR)	1367.5 | 459	1340 | 558	1925.0	0.972^#^	
BW <1000 grams, n (%)	13 (23)	16 (23)	0.0	0.997^*^	
Male, n (%)	18 (32)	34 (49)	3.735	0.053^*^	
SGA, n (%)	16 (29)	10 (15)	3.719	0.054^*^	
ACT, n (%)	48 (86)	59 (86)	0.001	0.974^*^	
Cesarean delivery, n (%)	41 (73)	35 (51)	6.560	0.010^*^	2.66 (1.25-5.60)
Advanced resuscitation, n (%)	9 (16)	13 (19)	0.163	0.686^*^	
Surfactant administration, n (%)	13 (23)	18 (26)	0.137	0.712^*^	
IMV, n (%)	15 (27)	20 (29)	0.074	0.785^*^	
BPD, n (%)	1 (2)	2 (3)	-	1^**^	
PIVH, n (%)	2 (4)	4 (6)	-	0.690^**^	
Hydrocephalus needing derivation, n (%)	1 (2)	2 (3)	-	1^**^	
CPL, n (%)	1 (4)	3 (5)	-	0.627^**^	
NEC, n (%)	0	4 (6)	-	0.127^**^	
Sepsis, n (%)	5 (9)	7 (10)	0.053	0.818^*^	
ROP, n (%)	3 (5)	5 (7)	-	0.730^**^	
Neonatal mortality, n (%)	7 (10)	8 (9)	0.139	0.709^*^	
Moderate to severe NDI, n (%)	2 (4)	5 (7)	-	0.458^**^	

Neurodevelopment assessment at 24 months of corrected age revealed that 5.6% (n=7) of the infants had moderate to severe NDI: 3.2% (n=4) had a GMDS-II global score <70 and 2.4% (n=3) were diagnosed with CP (two with GMFS level IV and one with level II). None had sensorineural deafness or severe visual impairment. Regarding chorionicity, 29% (n=2) of infants with moderate to severe NDI were MC-T and 71% (n=5) were BC-T, with no statistically significant difference between them. The two MC-T individuals were siblings affected by TTTS.

The evaluation of possible risk factors for moderate to severe NDI is represented in Table 2. Individuals with moderate to severe NDI were born at a lower GA (27 weeks vs. 31 weeks, p=0.022) and with lower BW (1360g vs. 945g, p=0.017) than those without moderate to severe NDI. They required more IMV and suffered from more prematurity-related morbidities, such as severe PIVH (p=0.036), moderate to severe BPD (p<0.001), and ROP ³ stage 2 (p<0.001).

**Table 2:** Characteristics and comparison of infants with and without moderate to severe NDI ACT – antenatal corticosteroid therapy; BPD – bronchopulmonary dysplasia; BW – birth weight; CPL – cystic periventricular leukomalacia; GA – gestational age; IMV – invasive mechanical ventilation; IQR – interquartile range; NDI – neurodevelopment impairment; NEC – necrotizing enterocolitis; PIVH – periventricular-intraventricular hemorrhage; ROP – retinopathy of prematurity; SGA – small for gestational age Categorical variable data are expressed in frequencies (n) and percentages (%) and analyzed using the *χ² test or **Fisher’s exact test, as appropriate, with respective odds ratio. The test value is Fisher’s exact test, which is not displayed, as it is not applicable. Continuous variable data are expressed in median and interquartile ranges and analyzed using the #Mann-Whitney U test. Statistical significance was considered when p-value <0.05.

	Infants without moderate to severe NDI (n=118)	Infants with moderate to severe NDI (n=7)	Test value	p-value*	OR (CI 95%)
GA (weeks) (median | IQR)	31 | 3	27 | 6	202.5	0.022^#^	
GA <28 weeks, n (%)	17 (14)	4 (57)	-	0.015^**^	7.92 (1.63-38.56)
BW (gram) (median | IQR)	1360 | 448	945 | 540	190.5	0.017^#^	
BW <1000 gram, n (%)	25 (21)	4 (57)	-	0.050^**^	
Monochorionicity, n (%)	54 (46)	2 (29)	-	0.458^**^	
Male, n (%)	47 (40)	5 (71)	-	0.126^**^	
SGA, n (%)	25 (21)	1 (14)	-	0.704^**^	
ACT, n (%)	102 (86)	5 (71)	-	0.265^**^	
Cesarean delivery, n (%)	71 (60)	5 (71)	-	0,704^**^	
Advanced resuscitation, n (%)	16 (14)	6 (86)	-	<0.001^**^	38.25 (4.32-338.92)
Surfactant administration, n (%)	25 (21)	6 (86)	-	<0.001^**^	22.32 (2.57-194.04)
IMV, n (%)	29 (24)	6 (86)	-	0.002^**^	18.41 (2.13-159.36)
BPD, n (%)	0 (0)	3 (43)	-	<0.001^**^	21.38 (3.54-129.13)
PIVH, n (%)	4 (3)	2 (29)	-	0.036^**^	11.4 (1.67-77.69)
Hydrocephalus needing derivation, n (%)	2 (2)	1 (14)	-	0.160^**^	
CPL, n (%)	3 (2.5)	1 (14)	-	0.208^**^	
NEC, n (%)	3 (2.5)	1 (14)	-	0.208^**^	
Sepsis, n (%)	10 (9)	2 (29)	-	0.135^**^	
ROP, n (%)	4 (3)	4 (57)	-	<0.001^**^	21.14 (3.94-113.51)
Fetal death of co-twin, n (%)	1 (1)	1 (14)	-	0.109^**^	

After performing an adjustment by logistic regression, none of the evaluated factors (GA, BW, chorionicity, ROP, PIVH, BPD, IMV, and advanced resuscitation) proved to be an independent risk factor for the presence of moderate to severe NDI. 

**Figure 2:**
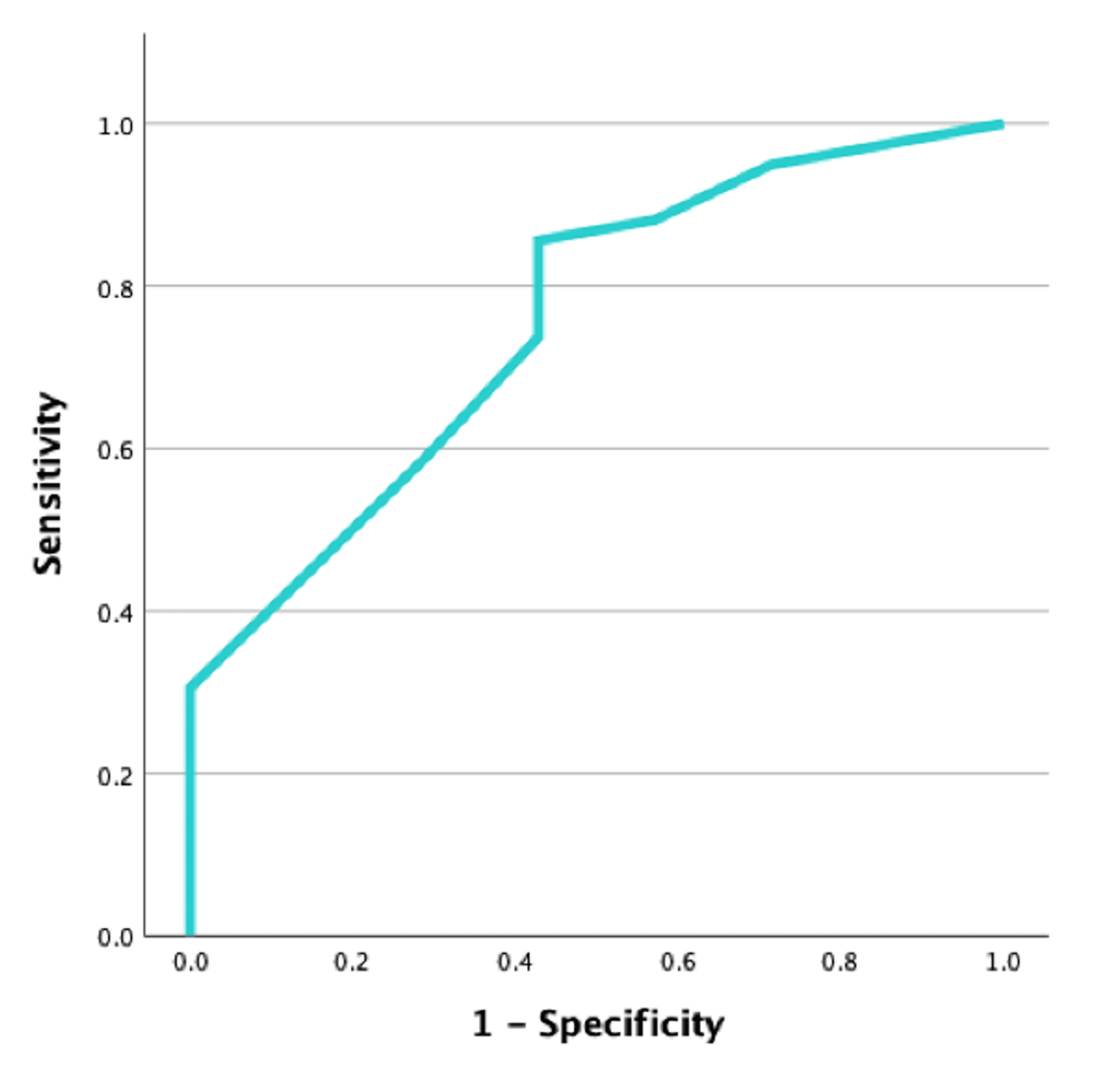
ROC curve for gestational age (AUC= 0.755, p=0.004; CI 95% 0.58-0.93) ROC – receiver operating characteristics; AUC – area under the curve

**Figure 3:**
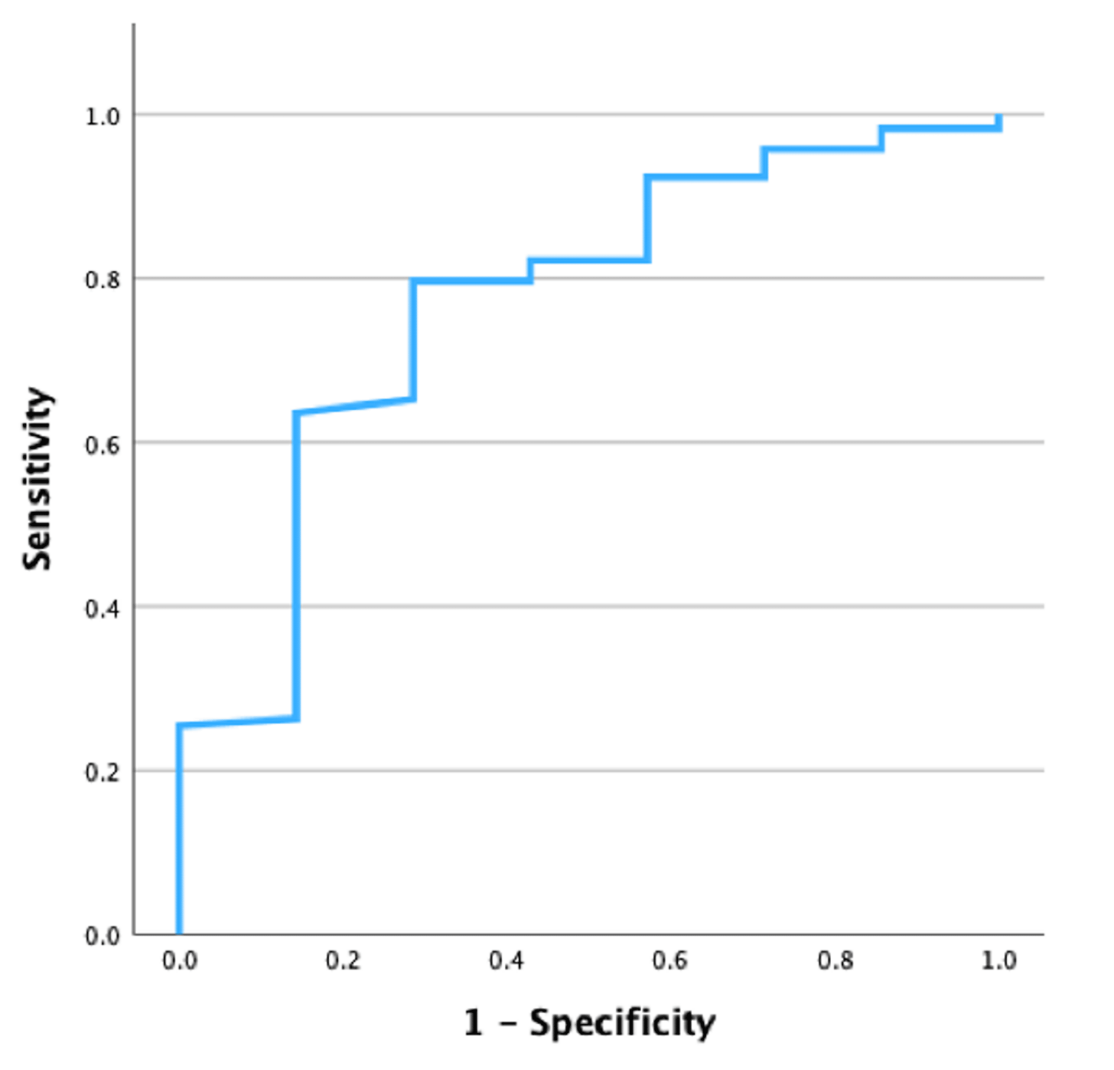
ROC curve for birth weight (AUC = 0.769, p=0.003; 95% CI 0.59-0.95) ROC – receiver operating characteristics; AUC – area under the curve

When we evaluated severe to moderate NDI in relation to GA, we verified that the cut-off of 27 weeks was a good predictor of the absence of moderate to severe NDI (AUC = 0.755; p = 0.004; CI 95% 0.58-0.93), with a sensitivity of 85.6% and specificity of 57.1% (Figure 2). Regarding BW, we verified that a cut-off of 1010 g was also a good predictor of the absence of moderate to severe NDI (AUC = 0.769; p = 0.003; CI 95% 0.59-0.95), with a sensitivity of 79.7% and specificity of 71.4% (Figure 3).

## Discussion

The continuous increase of multifetal pregnancies in the past decades, associated with increased risk of prematurity, has brought attention to the significance of evaluating sequelae and quality of life and not only focusing on survival [2,14].

The impact of chorionicity on the prevalence of neurological morbidity remains uncertain. Although several studies have investigated the relationship between chorionicity and neurological outcomes in twin pregnancies, their findings have been inconsistent [25].

In this study, we evaluate the NDI in multifetal pregnancies. We found no differences in moderate to severe NDI among the studied groups (MC-T and BC-T). This differs from what has been previously reported in the literature, where most studies attribute the higher rates of neurodevelopmental sequelae in MC-T to comorbidities associated with monochorionicity such as TTTS and intrauterine growth restriction [8,12,25].

Adedayo et al. described significantly higher rates of incidence of minor neurologic disability (15% vs 3%, p<0.05) and CP (8% vs 1%, p<0.05) in preterm MC-T than the gestational age-matched BC-T [12]. Minakami showed similar results, with an increased risk of adverse outcomes in MC-T associated with TTTS and discordant birth weights [26]. This was also reported in a study conducted in our institution in the previous decade, which revealed worse outcomes in MC-T compared to BC-T [27].

Our study reflects a different reality, that had already been stated in a systematic review by Yan et al. [25] The current findings may reflect the progress in the surveillance and follow-up programs of monochorionic pregnancies, particularly with early referral and treatment of TTTS. This could explain why, despite a 27% incidence of TTTS in our MC-T cohort (a known predictor of poor outcomes), the rates of severe to moderate NDI were comparable to those observed in BC-T.

Multifetal pregnancies have a higher morbidity, in association with prematurity, which is itself known as an important risk factor for NDI [1,7,28]. Our study revealed that it was possible to accurately predict the absence of moderate to severe NDI after 27 weeks, with GA under 27 weeks serving as an indicator of poorer outcomes. 

Apart from this, monochorionic pregnancies have been regarded as a higher risk for comorbidities due to risk factors and complications associated with chorionicity such as TTTS [25,29,30]. Therefore, in cases where these risk factors are manageable or treatable, namely, with fetoscopic laser surgery, the impact of chorionicity in MC-T may be mitigated, resulting in outcomes similar to those seen in BC-T [25].

Factors contributing to NDI are complex, as neurodevelopment is a continuous process shaped by the interaction between biological and environmental conditions. Thus, neurodevelopmental disorders tend to occur in children who have suffered an insult that compromises the normal maturity of the nervous system [25]. Given that MC-T are more vulnerable to various complications, close monitoring of these pregnancies is essential to identify and address factors associated with morbimortality and NDI, ultimately improving outcomes.

Our findings, showing the absence of differences in severe to moderate NDI in MC-T and BC-T, are probably related to a better surveillance program developed throughout the years. The precocious identification of chorionicity-related complications and early referral to adequate treatment, like laser surgery, certainly contributed to our results.

The main strengths of our study include a high follow-up rate, with neurodevelopmental assessments conducted in 87% of selected patients. These evaluations, performed by skilled professionals at specific developmental milestones, enhance the robustness of our results and represent the only established protocol of its kind in our country.

Nonetheless, the study also has some limitations. First, its retrospective nature and small sample size may affect the quality of the analysis. However, memory and registration biases are minimized, as the data for this population is systematically recorded from birth to discharge by some of the authors in the National Registry of Very Preterm Newborns database. Second, the lack of neurodevelopmental assessments beyond 24 months of age limits our ability to analyze longer-term outcomes and the impact of premature birth. Further studies, particularly prospective trials with long-term follow-up, are warranted to address these issues. 

## Conclusions

Our research did not identify chorionicity as an independent risk factor for adverse neurodevelopmental outcomes in preterm twins, provided that medical complications are promptly and effectively treated.

This highlights the importance of comprehensive and meticulous monitoring throughout the course of twin pregnancies, particularly in MC-T, which require heightened vigilance due to their unique risks and potential complications. Such proactive care plays a vital role in mitigating the challenges associated with preterm twin gestations, being essential to minimize the likelihood of neurodevelopmental impairment.
